# Enhanced rock weathering: biological climate change mitigation with co-benefits for food security?

**DOI:** 10.1098/rsbl.2017.0149

**Published:** 2017-04-05

**Authors:** David J. Beerling

**Affiliations:** Leverhulme Centre for Climate Change Mitigation, Department of Animal and Plant Sciences, University of Sheffield, Sheffield S10 2TN, UK

Under the Paris Agreement of the UN's 21st Conference of the Parties, over 100 nations signed up to the goal of keeping future warming within 2°C of pre-industrial levels, and ideally below 1.5°C. Yet anthropogenic CO_2_ emissions, mainly from combustion of fossil fuels, are now the highest they have been in human history, and 30% higher than 1990 [1]. Accumulation of CO_2_ and other human-caused greenhouse gases in the atmosphere has already driven global warming of approximately 1°C. If warming continues at the current rate, the aspirational target of 1.5°C will be out of reach within 30 years.

The urgent need for developing methods to extract CO_2_ from air (so-called negative emission technologies, NETs) that are safe and affordable, and that can be scaled-up to augment efforts to reduce CO_2_ emissions, is becoming increasingly well recognized and understood (e.g. [2]). Indeed, extensive modelling scenarios assessed by the Intergovernmental Panel on Climate Change that give us more than a 50% chance of limiting warming to less than 2°C assume substantial CO_2_ extraction is achievable with bioenergy crops in combination with carbon capture and storage (BECCS) in the second half the 21st Century [3]. However, major assumptions about land availability, feasibility at scale, and costs involved raise doubt about the promise and effectiveness of BECCS [4,5].

A range of potential techniques for extracting CO_2_ from the atmosphere is being investigated that afford opportunities for mitigating and ameliorating climate change (figure 1), each of which also needs to be understood in terms of feasibility, cost and acceptability [6,7]. The papers in this mini-series address an underdeveloped NET, enhanced rock weathering, with a particular focus on croplands managed for food production and bioenergy. Weathering is a slow natural process removing CO_2_ from the atmosphere on long timescales of a million years or more. During weathering, silicate rocks are chemically broken down to release base cations and generate bicarbonate, which is ultimately transferred to the oceans leading to carbonate precipitation on the seafloor. These processes can be accelerated by amending soils with crushed calcium and magnesium-bearing silicate rocks with a high reactive surface area to deliver effective carbon sequestration in soils and the oceans [8,9]. Enhanced weathering is also co-deployable with forestry and crops used in BECCS, enhancing its carbon sequestration potential and reducing costs. Reduced atmospheric CO_2_, in combination with the production of soluble alkalinity from weathered rocks, can help reduce ocean acidification to protect coral reefs and marine fisheries [8,9].

**Figure 1.**
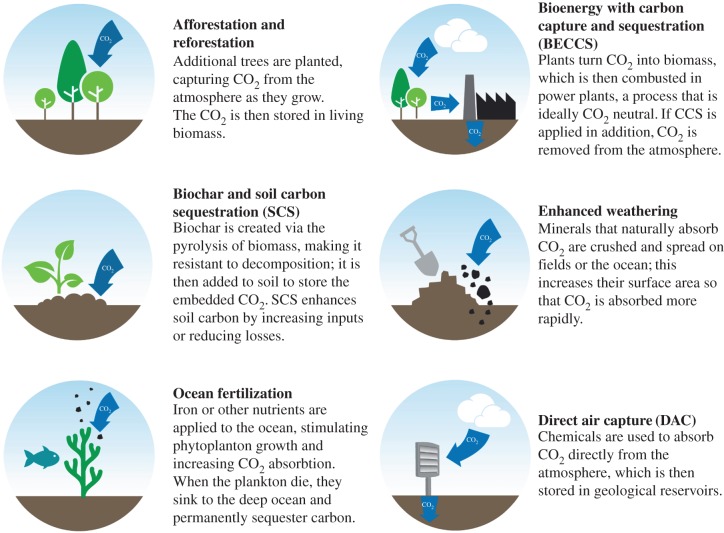
Six common categories of negative emissions strategies for extracting CO_2_ from the atmosphere. Reproduced with permission from [7].

Kantola *et al.* [10] deal with the opportunities presented by row crop agricultural production of food and bioenergy crops for enhancing rock weathering. They highlight mechanisms by which enhanced weathering on agricultural lands could combat soil acidification and nitrogen loss while providing plant-essential nutrients, two negative consequences of intensive cropland farming. Uncertainties in the long-term effects on soils and crops remain and can only be addressed through long-term experiments and field trials investigating feasibility and effectiveness. Nevertheless, with nearly 11% of the terrestrial surface intensively managed for crop production, enhanced weathering could offer an opportunity to employ these soils to sequester atmospheric carbon at scale within a decade or two, while benefitting crop production. Additionally, this would use land already in production, thereby avoiding potential land use conflicts.

Edwards *et al.* [11] provide a complementary tropical cropland perspective, focusing on the potential for deployment of enhanced weathering across over 680 million hectares of tropical agriculture, with the warm climates and productive crops substantially accelerating weathering. They identify potential co-benefits including decreased soil acidification, reduced heavy metal toxicity and increased phosphorus-supply of highly weathered nutrient-poor tropical soils promoting higher crop yields. This may have the effect of sparing forest for conservation, and reducing cultural eutrophication. Recycling the substantial annual global production of silicate waste resulting from human activities [12] would minimize the impacts of mining operations on the environment, including deforestation, and reduce energy requirements for crushing and transporting silicates. Negative consequences identified [11] include erosion of silicates into rivers and coral reefs that might increase inorganic turbidity, sedimentation and pH, with unknown impacts for biodiversity. They conclude by outlining a research agenda for responsibly unlocking the potential of the tropics for carbon capture by enhanced weathering, including assessment of the potential impacts on human health, farmland, forest, stream-water chemistry and biodiversity.

Questions concerning the spatial scale of roll-out necessary to affect atmospheric CO_2_, climate and ocean chemistry on decadal to century timescales can only be addressed through Earth system modelling [8,9]. Central to these large-scale Earth system issues are robust numerical models describing the geochemical weathering of crushed silicates by climate in combination with the rooting systems of crop plants and their associated soil microbes. In this context, Taylor *et al.* [13] review how current models represent the geochemical processes describing the soil weathering CO_2_ sink. They highlight the need to bridge the gap between the current generation of weathering models that typically neglect agricultural processes and agricultural models simulating how land management practices govern cropland soil chemistry and greenhouse gas emissions that neglect weathering. Land use history and fertilizers are key drivers of the physico-chemical characteristics of agricultural soils, including pH. Capturing these interactions with crushed silicates will be an important development of soil weathering models if they are to provide powerful and flexible research tools for assessment of rates of enhanced weathering, nutrient release, pH change and carbon capture.

Meysman & Montserrat [14] shift the focus to the marine realm by considering the potential for carbon capture by applying crushed silicates directly into coastal environments. The idea is that deliberately introducing fast-weathering silicate minerals onto coastal sediments releases alkalinity into the overlying waters, thus creating a coastal CO_2_ sink. As they point out, the concept is still at an early stage and dedicated experiments need to (i) better constrain the weathering rate under *in situ* conditions, and (ii) evaluate ecosystem impacts arising from the liberation of weathered chemical products.

Ultimately, the public perception of any NET option may prove to be as important as the underlying scientific evidence that builds the case for deployment to combat climate change. If policymakers and governments propose deployment, there has to public trust and acceptance of the technology involved. Social science engagement is therefore critical to understanding how society will perceive different NET options [15]. How might public groups in the UK, North America and non-western countries, respond to and perceive potential risks and benefits of possible enhanced weathering strategies as innovative responses to the climate change problem? Pidgeon & Spence [16] report the first UK-wide assessment of the public's social perception of enhanced terrestrial weathering. Their analyses provide an important baseline for determining how this may change as the technology gains prominence and for comparing the UK public with other parts of the world. Encouragingly, it appears the public generally agree that scientists should be able to conduct small-scale trials into enhanced weathering, provided there is scientific independence, strict monitoring, risk minimization and transparency of results.

Lawford-Smith & Currie [17] address some of the thorny ethical issues associated with developing negative emissions options. They analyse the well-rehearsed moral hazard argument in relation to questions of blame and responsibility for removing the onus on developed countries to reduce fossil fuel emissions. Would large-scale effective NETs deployed in the future lead to less mitigation today [18]? Might, for example, it encourage society to think CO_2_ emissions can exceed ‘safe’ limits in the near-term with the promise that excess carbon will pulled out of the atmosphere later? Given current lack of depth in our understanding of the effectiveness of NETs, a safer working assumption might be that they may not be deployable this century, forcing the urgency of deeper near-term emission cuts to avoid locking in the worst effects of future climate change.

Collectively, the papers in this mini-series suggest that enhanced weathering has promise in providing climate and food security by capturing carbon and improving crop yields, while decreasing fertilizer and pesticide usage and costs. The collection is not in any way intended to be a comprehensive treatment of the topic but rather to represent an introduction to some of the key issues with an emphasis on biological interactions; detailed treatment of enhanced weathering is given elsewhere [19]. The topic is, however, the focus of the newly established international Leverhulme Centre for Climate Change Mitigation (http://www.lc3m.org/). The new Centre aims to deliver transformative understanding of all aspects of enhanced weathering with croplands as a strategic NET, including its technical, environmental, economic and social viability, as highlighted in a recent *Nature Geoscience* editorial [20]. It aims to revolutionize climate change mitigation by linking it to the substantive co-benefit of delivering resource-efficient sustainable food security. Moving towards these goals will require fundamentally understanding our ability to manipulate food/bioenergy production systems to drive biogeochemical cycles that positively affect global CO_2_, climate and ocean chemistry—a formidable challenge that underpins some climate change mitigation strategies required by the Paris Agreement.

## References

[1] Le Quere C et al. 2016 Global carbon budget 2016. Earth Syst. Sci. Data 8, 605–649. (10.5194/essd-8-605-2016)

[2] National Research Council. 2015 Climate intervention. Carbon dioxide removal and reliable sequestration. Washington, DC: The National Academies Press.

[3] Intergovernmental Panel on Climate Change. 2014 Climate Change 2014: Mitigation of Climate Change (eds O Edenhofer *et al*.) New York, NY: Cambridge Univ. Press.

[4] Fuss SF et al. 2014 Betting on negative emissions. Nat. Clim. Change 4, 850–853. (10.1038/nclimate2392)

[5] Anderson K 2015 Talks in the city of light generate more heat. Nature 528, 437 (10.1038/528437a) 26701018

[6] Smith P et al. 2016 Biophysical and economic limits to negative CO_2_ emissions. Nat. Clim. Change 6, 42–50. (10.1038/nclimate2870)

[7] Minx JC, Lamb WF, Callaghan MW, Bornmann L, Fuss S 2017 Fast growing research on negative emissions. Environ. Res. Lett. 12, 035007 (10.1088/1748-9326/aa5ee5)

[8] Köhler P, Hartmann J, Wolf-Gladrow DA 2010 Geoengineering potential of artificially enhanced silicate weathering of olivine. Proc. Natl Acad. Sci. USA 107, 20 228–20 233. (10.1073/pnas.1000545107) PMC2996662 21059941

[9] Taylor LL et al. 2016 Enhanced weathering strategies for stabilizing climate and averting ocean acidification. Nat. Clim. Change 6, 402–406. (10.1038/nclimate2882)

[10] Kantola IB, Masters MD, Beerling DJ, Long SP, DeLucia EH 2017 Potential of global croplands and bioenergy crops for climate change mitigation through deployment for enhanced weathering. Biol. Lett. 13, 20160714 (10.1098/rsbl.2016.0714) 28381630 PMC5414685

[11] Edwards DP, Lim F, James RH, Pearce CR, Scholes J, Freckleton RP, Beerling DJ 2017 Climate change mitigation: potential benefits and pitfalls of enhanced rock weathering in tropical agriculture. Biol. Lett. 13, 20160715 (10.1098/rsbl.2016.0715) 28381631 PMC5414686

[12] Renforth P, Washbourne CL, Taylder J, Manning DAC 2011 Silicate production and availability for mineral carbonation. Environ. Sci. Technol. 45, 2035–2041. (10.1021/es103241w) 21332128

[13] Taylor LL, Beerling DJ, Quegan S, Banwart SA 2017 Simulating carbon capture by enhanced weathering with croplands: an overview of key processes highlighting areas of future model development. Biol. Lett. 13, 20160868 (10.1098/rsbl.2016.0868) 28381633 PMC5414688

[14] Meysman FJR, Montserrat F 2017 Negative CO_2_ emissions via enhanced silicate weathering in coastal environments. Biol. Lett. 13, 20160905 (10.1098/rsbl.2016.0905) 28381634 PMC5414690

[15] Wright MJ, Teagle DAH, Feetham PM 2014 A quantitative evaluation of the public response to climate engineering. Nat. Clim. Change 4, 106–110. (10.1038/nclimate2087)

[16] Pidgeon NF, Spence E 2017 Perceptions of enhanced weathering as a biological negative emissions option. Biol. Lett. 13, 20170024 (10.1098/rsbl.2017.0024) 28381635 PMC5414695

[17] Lawford-Smith H, Currie A 2017 Accelerating the carbon cycle: the ethics of enhanced weathering. Biol. Lett. 13, 20160859 (10.1098/rsbl.2016.0859) 28381632 PMC5414687

[18] Anderson K, Peters G 2016 The trouble with negative emissions. Science 354, 182–183. (10.1126/science.aah4567) 27738161

[19] Hartmann J, West AJ, Renforth P, Köhler P, La Rocha CLD, Wolf-Gladrow DA, Dürr HH, Scheffran J 2013 Enhanced chemical weathering as a geoengineering strategy to reduce atmospheric carbon dioxide, supply nutrients, and mitigate ocean acidification. Rev. Geophys. 51, 113–149. (10.1002/rog.20004)

[20] Anon. 2016 A step up for geoengineering [editorial]. Nat. Geosci. 9, 855 (10.1038/ngeo2858)

